# Supplementary material for: Microbial Sharing between Pediatric Patients and Therapy Dogs during Hospital Animal-Assisted Intervention Programs
Source: Microorganisms. 2021 May 13;9(5):1054. doi: 10.3390/microorganisms9051054 (PMC8153335; doi:10.3390/microorganisms9051054)
Supplement: Supplementary file 1 [file microorganisms-09-01054-s001.zip › microorganisms-1195111-supplementary.pdf]

<u>Supplementary Materials</u>

Supplemental Methods: Detailed DNA Extraction Protocol

The Puritan swab samples were thawed, and 0.5 μL of Ready-Lyse Lysozyme (Epicentre Biotechnologies, Madison, WI) was added to each tube and incubated for 1 h with shaking at 600 rpm and 37°C. The swab was re- moved, placed into a spin basket, and centrifuged for 1 min at 9,400 × g to extract any remaining liquid. The sample was then added to a glass bead tube (0.5 mm; MO BIO, Carlsbad, CA) and vortexed for 10 min at maximum setting. The samples were then incubated in a heat block for 30 min at 65°C and 600 rpm, followed by ice for 5 min and a brief spin. A 150 μL of Protein Precipitation Buffer (Epicentre Biotechnologies, Madison, WI) was added, and the samples were vortexed briefly, then centrifuged at 22,000 × g for 10 min. The supernatant was removed and the protein pellet was discarded. The supernatant was mixed with 500 μL isopropanol and inverted to mix. The mixture was added to a spin column from the Genomic DNA Isolation Kit (Life Technologies, Grand Island, NY), and the remaining steps were followed according to manufacturer's protocol. The samples were eluted with 50 μL Elution Buffer (Life Technologies, Grand Island, NY).

Supplemental Table 1A: 16S rRNA gene Sequencing Library Results

Read Counts				
**Sample**	**Min**	**Mean**	**Median**	**Max**
Total	1	10736	13342	21890
Child N	164	11639	14100	19430
Dog All	711	12559	14448	21890
N	711	9315	8598	21890
MO	4980	14144	14989	16502
I	941	11389	13140	17373
R	12745	15207	14967	18503
Environment	1	12051	13048	16109
Field Blank	520	4676	4307	10064
Controls				
Extraction	189	2357	2296	5207
Sequence	1	26.2	8	117
Mock	1	4.5	3.5	10
**Total DNA Concentration (ng/ul)**				
Total	0.04	8.517	3	41.39
Child N	0.05	5.434	2.25	31.67
Dog All	0.16	11.001	7.05	41.39
N	0.16	3.92	0.975	18.5
MO	0.6	18.8	19	41.1
I	0.18	3.19	2.14	9.46
R	1.49	16.9	13.9	41.39
Environment	0.07	19.77	19.98	38.82
Field Blank	0.2	0.7717	0.745	1.61
Controls				
Extraction	0.06	0.327	0.31	0.67
Sequence	0.08	0.2267	0.16	0.7
Mock	0.04	0.105	0.08	0.22
**qPCR 16S gene copies (/ul DNA)**				
Total	43	1.42e6	4570	6.26e7
Child N	111	16073	2470	2.58e5
Dog All	208	1.41e6	10850	6.26e7
N	208	7630	1730	70100
MO	1080	1.72e6	2.24e5	2.52e7
I	314	10782	3940	77600
R	838	3.69e6	95000	6.26e7
Environment	185e5	7.94e6	5.91e6	2.45e7
Field Blank	88	481	405	1040
Controls				
Extraction	56.6	460	273	1400
Sequence	61.9	344	293	729
Mock	42.8	233	181	475

Supplemental Table 1B: Microbial Community Sequencing Library Decontamination

Decontamination Stage	Number Contaminants (% removed)	Common Genera	Prevalence
Starting Taxa	**14183 total ASVs**		
Sequencing Controls	166 contaminants (1.17%)	*Corynebacterium*, *Sphingomonas*, *Streptococcus*, *Bacillus*	832nd most abundant
Extract Controls	149 contaminants (1.06%)	*Corynebacterium*, *Streptococcus*, *Staphylococcus* *Sphingomonas*	591st most abundant
Field Blanks	188 contaminants (1.36%)	*Corynebacterium, Streptococcus, Staphylococcus* *Sphingomonas*	705th most abundant
Final Taxa	**13680 total ASVs** (3.55%)		

Using ‘decontam’ package

Supplemental Table 2: AAI Behavioral Observations and Patient-Therapy Dog Contact Scores

**Observed Interaction Behaviors**	**Patients Observed**
N *	46
Total time spent with dog, in minutes<br>mean (range)	 14 (2-42)
Interact with Dog N (%)	44 (95.7)
Sat on Floor N (%)	28 (61.9)
Touch Head N (%)	43 (93.5)
Touch Back N (%)	27 (58.7)
Touch Belly N (%)	13 (28.2)
Touch Paws N (%)	11 (23.9)
Feed Dog N (%)	24 (52.2)
Walk Dog N (%)	10 (21.7)
Kiss Dog N (%)	2 (4.3)
Hug Dog N (%)	5 (10.9)
**Contact Score Level Calculation**	
Interact Score+ mean (range)	7.89 (0-17)
Interact Score x Time ^<br>mean (range)<br>median (IQR)	 127 (0-403)<br>108 (56.25-197.75)
Contact Score Level<br>High (≥median) N (%)<br>Low (<median) N (%)	 25 (51%)<br>24 (49%)

* observations not recorded for 3 patients
+ score based on total tally of individual behaviors, weighted based on closeness of contact: 1 point for interact with dog, walk dog, and sat on floor, 2 points touches, 3 points for feed, hug, or kiss dog.
^ score multiplied by total time with dog

Supplemental Table 3: Relative Abundance by Host and Site by Phyla and Genus
(Taxa > 3% total abundance)

Taxon (Phyla & Genus)	Patient Nasal	Dog Nasal	Dog Oral	Dog Perineal	Dog Inguinal	Environment
Actinobacteria	0.0483	0.0330	0.0432	0.2035	0.0197	0.0522
Corynebacterium	0.1130	0.0330	0.0922	0.2035	0.0431	0.1968
Micrococcus						0.0313
Actinomyces						
Bacteroidetes	0.0137	0.0213	0.0893	0.0704	0.0236	0.0248
Porphyromonas		0.0602	0.1604	0.1065	0.0673	
Capnocytophaga			0.0883			
Bacteroides				0.0734		
Prevotella				0.0312		0.0365
Firmicutes	0.0667	0.1147	0.0212	0.0360	0.0341	0.0633
Staphylococcus	0.2714	0.2886	0.0333	0.0649	0.0453	0.0465
Streptococcus	0.1834	0.0411		0.0952	0.0432	0.1011
Faecalibacterium						0.0688
Blautia				0.0433		
Alloiococcus	0.0382					
Lactobacillus						0.0369
Dorea				0.0317		
Fusobacteria		0.0120	0.0415	0.0370	0.0103	
Fusobacterium			0.0415	0.0370		
Proteobacteria	0.0368	0.0832	0.0559	0.0420	0.0191	0.0204
Moraxella	0.0572	0.1927	0.0396	0.0386		
Conchiformibius		0.0462	0.1879			
Lautropia			0.0741			
Campylobacter				0.0648		
Escherichia				0.0435		
Pantoea						0.0427
Neisseria			0.0408			
Sphingomonas					0.0349	
Pseudomonas					0.0345	
Spirochaetes			0.0274			

Supplemental Figure 1: Absolute Abundance of Key *Staphylococcus* Species

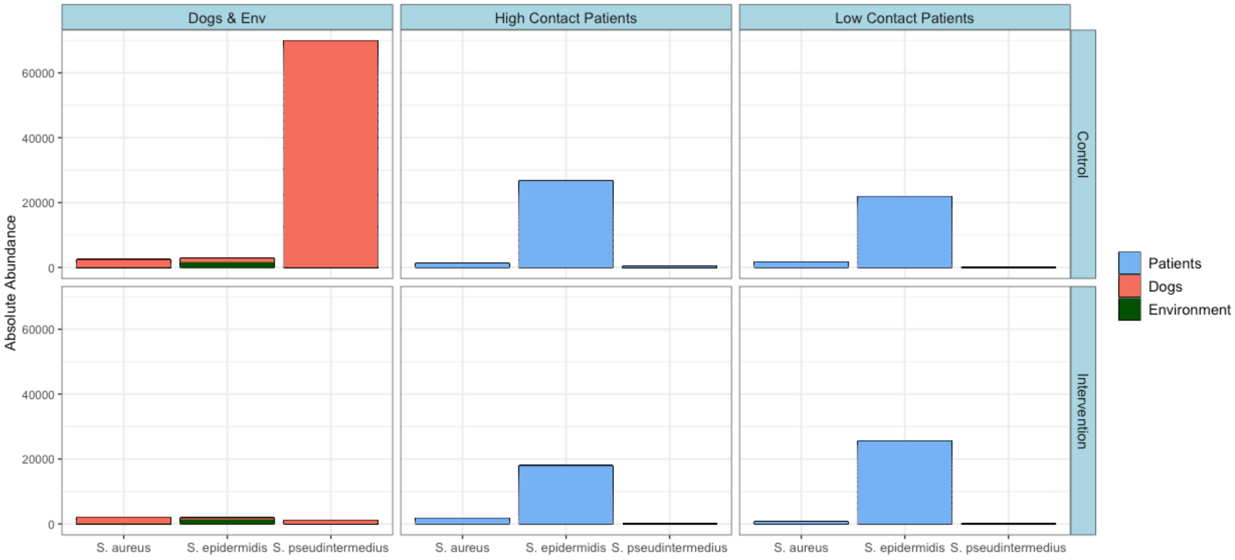

Supplemental Figure 2: Alpha Diversity Curves by Host

A. Total Taxa

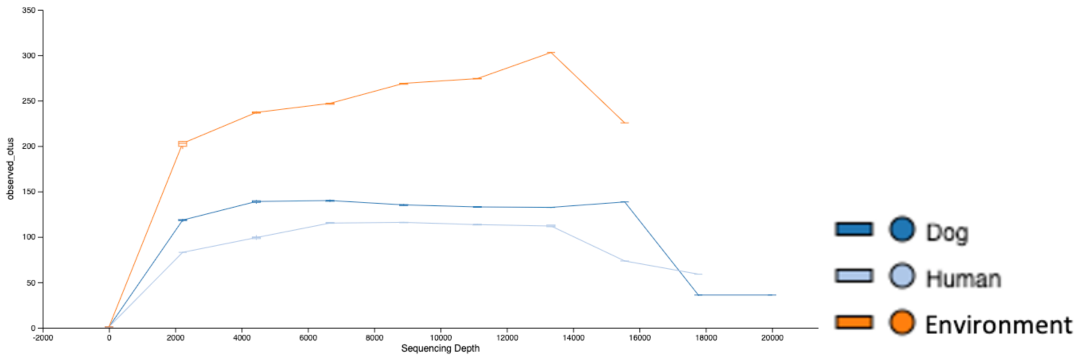

B. Shannon Diversity

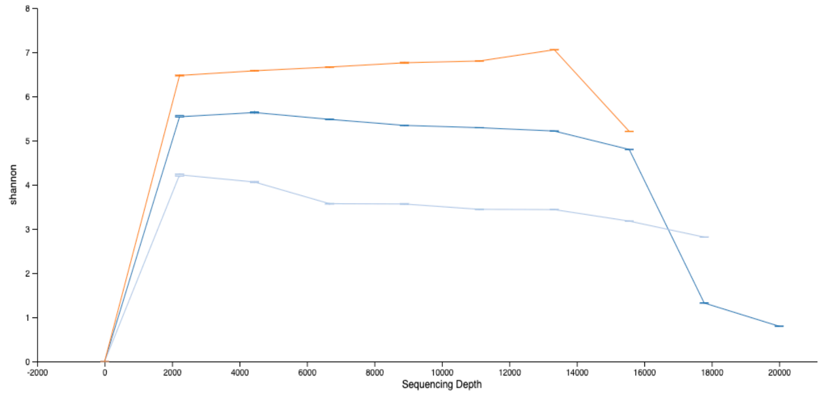

C. Faith's Phylogenetic Diversity

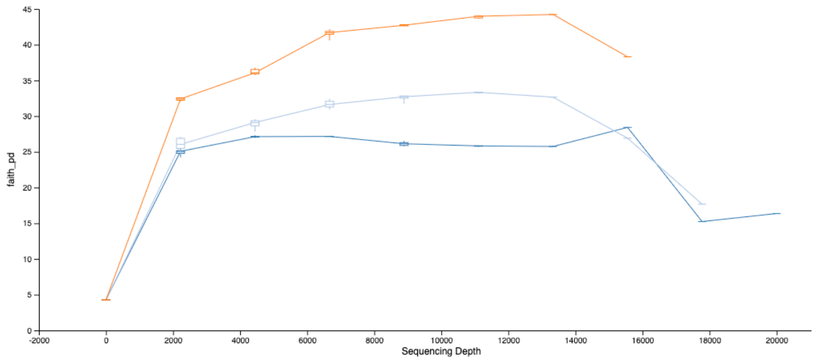

D. Number of Samples

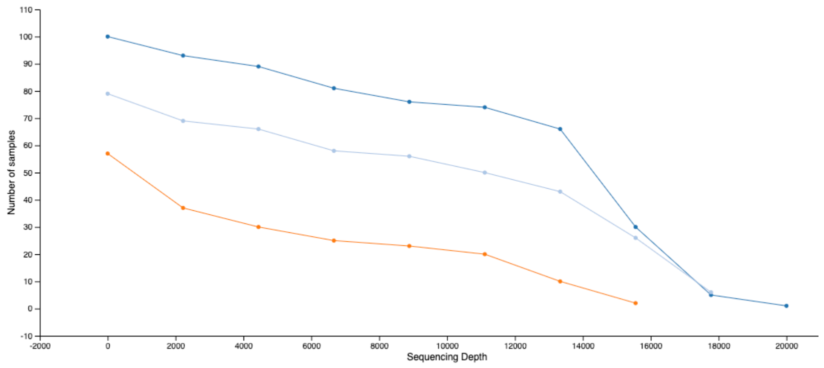

Supplemental Figure 3: Beta Diversity Principle Coordinates Analysis Plots

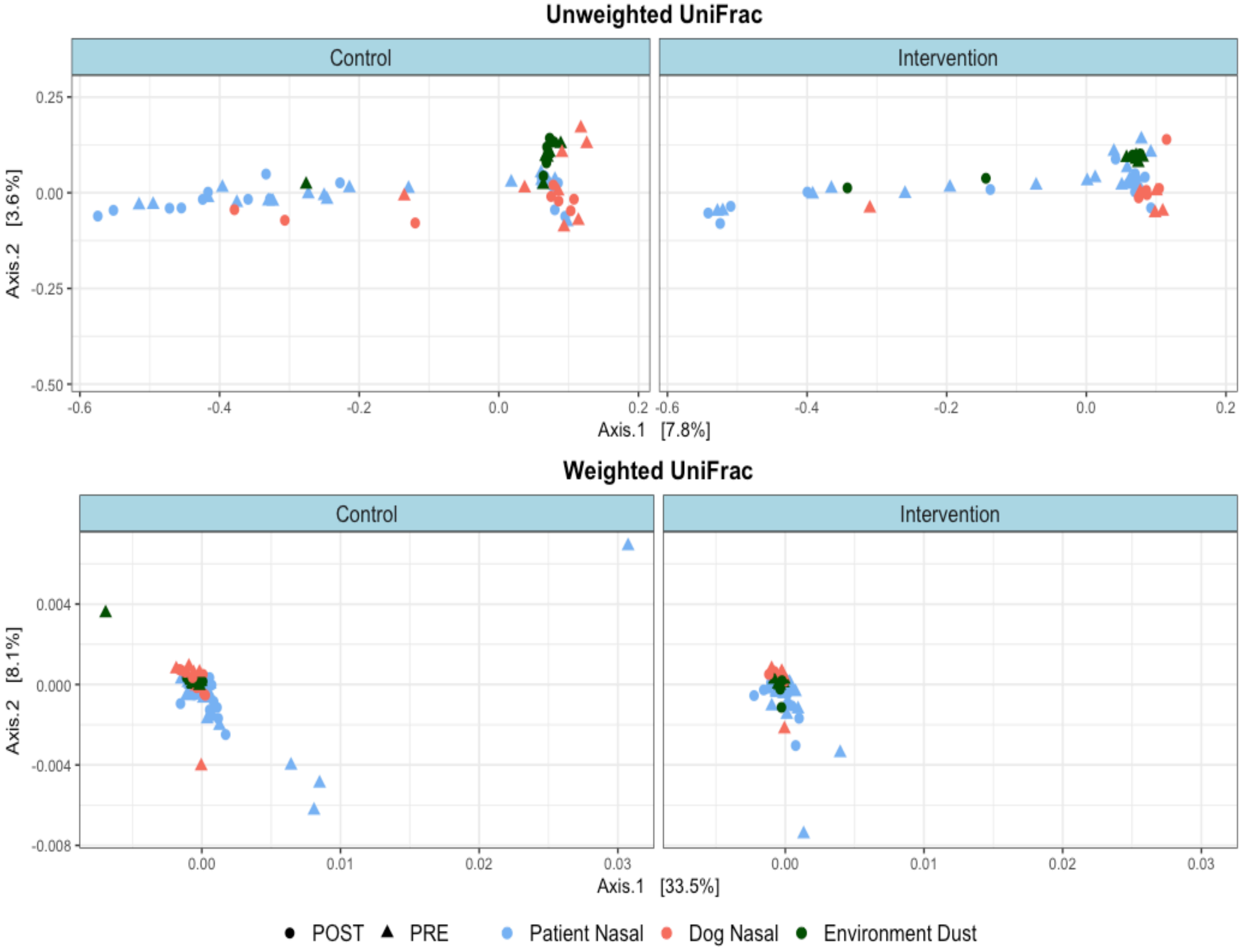

Supplemental Figure 4: Graphical Example for Calculations of Beta Distance in Figure 4

A. Kid-Kid Distance Example

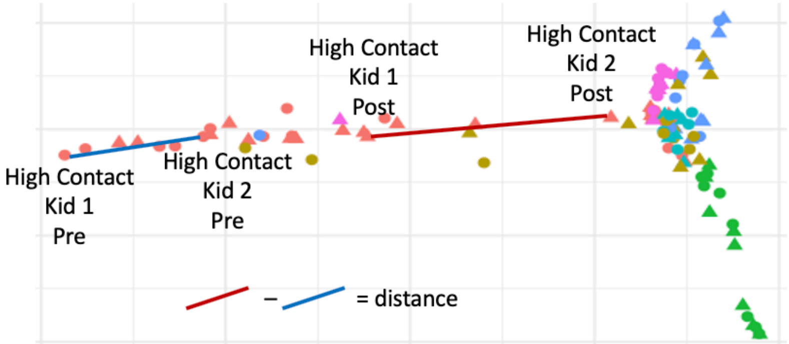

B. Kid-Dog Distance Example

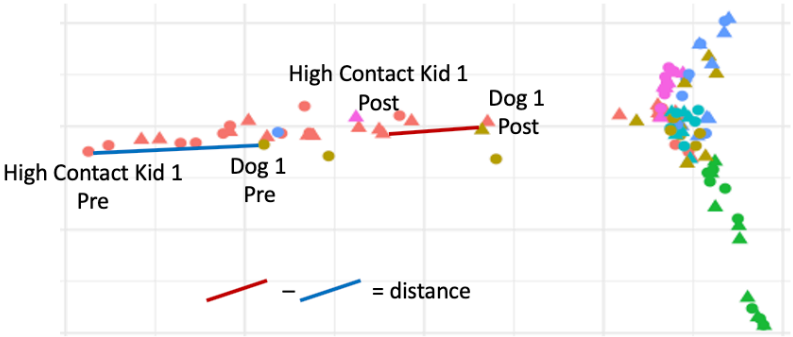

Supplemental Figure 5: Visit Type and Contact Level in Microbial Composition Differences Between Patients and Between Patients and Therapy Dogs

A. Unweighted UniFrac Distance

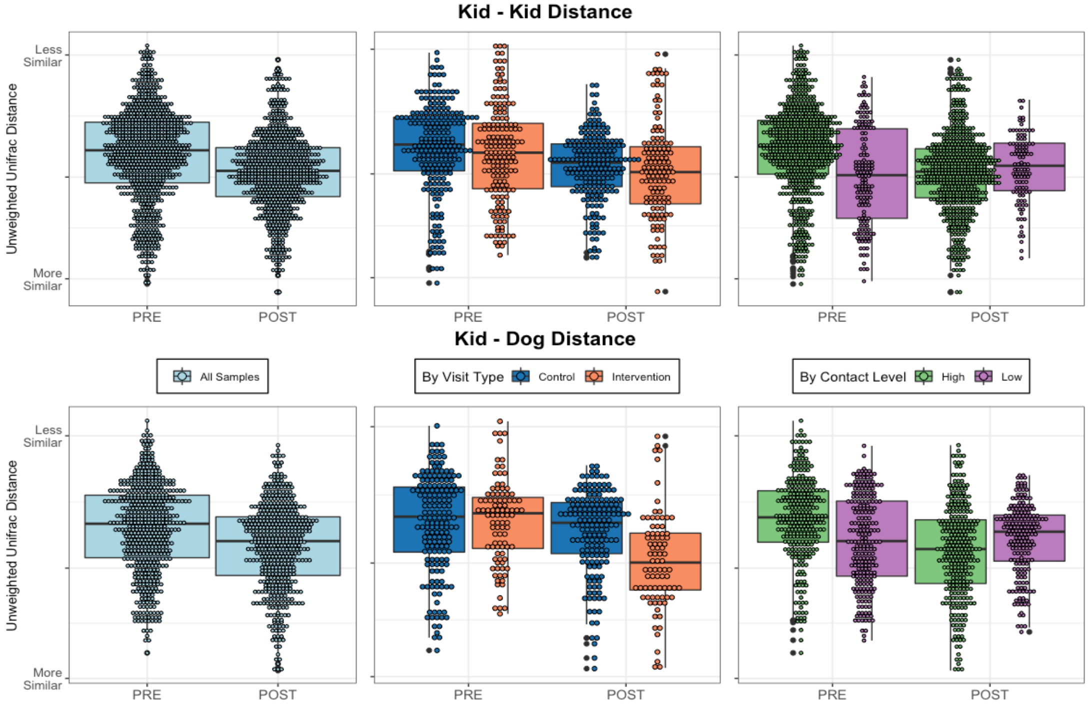

B. Weighted UniFrac Distance

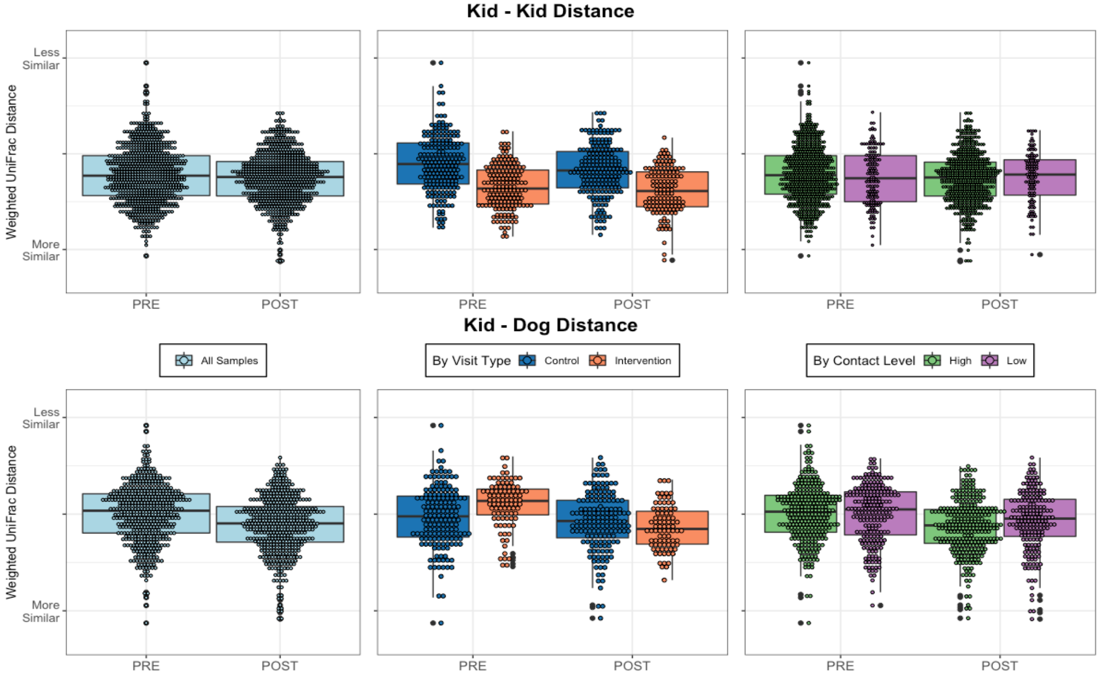

Supplemental Table 4: Microbial Composition Differences between Patients and Between Patients and Therapy Dogs Before the Visit Compared to After Visit, Based on Visit Type and Contact Score

Results for Beta Distance via PERMANOVA model

Group	Metric	Kid-Kid Distance		Kid-Dog Distance	
		F-statistic	p	F-statistic	p
**Unadjusted Effect Pre versus Post**					
All Samples	Unweighted UniFrac	47.068	**0.0001**	38.168	**0.0001**
	Weighted UniFrac	19.33	**0.0001**	36.378	**0.0001**
Control Visits	Unweighted UniFrac	15.926	**0.0002**	2.294	0.128
	Weighted UniFrac	17.78	**0.0001**	4.274	0.025
Intervention Visits	Unweighted UniFrac	8.737	*0.003*	35.188	**0.0001**
	Weighted UniFrac	1.049	0.313	44.689	**0.0001**
High Contact	Unweighted UniFrac	89.538	**0.0001**	82.758	**0.0001**
	Weighted UniFrac	16.017	**0.0001**	28.701	**0.0001**
Low Contact	Unweighted UniFrac	2.418	0.118	0.067	0.829
	Weighted UniFrac	0.474	0.514	9.766	*0.001*
**Multivariate Adjusted Effect**					
Pre versus Post visit	Unweighted UniFrac	47.642	**0.0001**	38.626	**0.0001**
	Weighted UniFrac	20.962	**0.0001**	36.422	**0.0001**
Contact Level	Unweighted UniFrac	10.317	**0.0002**	2.245	0.135
	Weighted UniFrac	6.691	**0.0003**	0.829	0.328
Visit Type	Unweighted UniFrac	1.325	0.263	4.728	0.011
	Weighted UniFrac	58.887	**0.0001**	2.138	0.094

PERMANOVA FDR-corrected **p<0.001**, *p 0.01-0.001*

Unadjusted Effect = difference in microbial composition distance between patients in pre-visit samples compared to microbial composition distance between patients in post-visit samples (kid-kid distance), or significant difference in microbial composition distance between patients and therapy dogs in pre-visit samples compared to microbial composition distance between patients and therapy dogs in post-visit samples (kid-dog distance) within each exposure group.

Example interpretation – In control visits, there is a significant difference in the microbial composition between patients before the visits compared to after the visit (p=0.0002 unweighted, 0.0001 weighted)

Adjusted Effect = independent effect of each exposure of the microbial composition distance between patients (kid-kid distance) or microbial composition distance between patients and therapy dogs (kid-dog distance).

Example interpretation – There is a significant difference in the microbial composition between patients with high contact compared to microbial composition between patients with low contact, independent of collection time (pre versus post) or visit type (control versus intervention) (p=0.0002 unweighted, 0.0003 weighted)
